# Specialist Palliative Care for Patients with Cancer: More Than End-of-Life Care

**DOI:** 10.3390/cancers15143551

**Published:** 2023-07-09

**Authors:** Craig Gouldthorpe, Jenny Power, Amy Taylor, Andrew Davies

**Affiliations:** 1 School of Medicine, Trinity College Dublin, DRW RY72 Dublin, Ireland; 2 Our Lady’s Hospice & Care Services, DRW RY72 Dublin, Ireland; 3 School of Medicine, University College Dublin, DRW RY72 Dublin, Ireland

**Keywords:** cancer, palliative care, holistic care, supportive care

## Abstract

Palliative care has traditionally focused on end-of-life care for patients with advanced cancer. This has since expanded to include symptom management and quality-of-life improvement from the moment of cancer diagnosis. Specialist palliative care teams work across community and inpatient settings and focus on dealing with complex problems whilst supporting healthcare colleagues in providing generalist palliative care. This article will outline the principles of palliative care, models of palliative care delivery, the distinctions between palliative care and supportive care, and the role of specialist palliative care in cancer survivorship.

## 1. Introduction

Palliative care has always been strongly associated with the care of patients with cancer. Whilst this originally focused on end-of-life care for patients with advanced cancer, the focus has shifted to include symptom management and quality-of-life improvement across the cancer trajectory and for patients with non-cancer conditions. Indeed, palliative care is deemed an essential component of modern oncology by organisations such as the American Society for Clinical Oncology (ASCO) and the European Society for Medical Oncology (ESMO) [1,2].

This article will highlight the current role(s) of palliative care within oncology and how these roles may change with the expectation of more patients “living with cancer” (and for longer) and “living beyond cancer” (i.e., long-term cancer survivors) as a result of advances in oncological treatments [3]. It will also highlight the similarities and differences between palliative care and supportive care and the role that palliative care teams play in supporting oncology and supportive care teams.

## 2. Definition of Palliative Care

The International Association for Hospice and Palliative Care (IAHPC) defines palliative care as “the active holistic care of individuals across all ages with serious health-related suffering due to severe illness and especially of those near the end of life. It aims to improve the quality of life of patients, their families and their caregivers” [4]. This definition was supported by a series of additional characteristics (Box 1), including that palliative care “is applicable throughout the course of an illness, according to the patient’s needs” and “is provided in conjunction with disease-modifying therapies whenever needed”. These latter points are especially relevant for patients with cancer.

Box 1 **Characteristics of palliative care [4].**

Palliative care:

▘ Includes the prevention, early identification, comprehensive assessment, and management of physical issues, including pain and other distressing symptoms, psychological distress, spiritual distress, and social needs. Whenever possible, these interventions must be evidence-based.
▘ Provides support to help patients live as fully as possible until death by facilitating effective communication, helping them and their families, determine the goals of care.
▘ Is applicable throughout the course of an illness, according to the patient’s needs.
▘ Is provided in conjunction with disease-modifying therapies whenever needed.
▘ May positively influence the course of the illness.
▘ Intends neither to hasten nor postpone death, affirms life, and recognizes dying as a natural process.
▘ Provides support to the family and caregivers during the patient’s illness, and in their own bereavement.
▘ Is delivered recognizing and respecting the cultural values and beliefs of the patient and family.
▘ Is applicable throughout all healthcare settings (places of residence and institutions) and in all levels (primary to tertiary).
▘ Can be provided by professionals with basic PC training.
▘ Requires specialist PC with a multi-professional team for referral of complex cases.

## 3. Specialist Palliative Care versus General Palliative Care

Palliative care services should be delivered according to levels of increasing expertise, and it is often divided into three levels: a palliative care approach, generalist palliative care, and specialist palliative care [5]. A palliative care approach assumes that all healthcare professionals have basic palliative care competencies and can integrate palliative care principles (see Box 1) into non-specialist settings [5]. Generalist palliative care is delivered by healthcare professionals within primary and secondary care, such as general practitioners and oncologists, who have undertaken additional studies and gained additional knowledge of palliative care [5]. Specialist palliative care refers to care provided by healthcare professionals with specific education, training, and experience in palliative care, whose primary role involves delivering such care [6]. Many of the characteristics of palliative care mirror the features of good holistic care and are routinely employed by the different members of an oncology multidisciplinary team. Indeed, most palliative care is provided, and will continue to be provided, by non-specialists due to resource constraints.

Thus, the main roles of specialist palliative care services are to provide input into complex problems, and to educate and support colleagues in providing optimal palliative care approaches and generalist palliative care [6]. Importantly, specialist palliative care services need to continue to develop the evidence base for palliative and end-of-life care since many contemporary interventions are based on expert opinion (anecdote) rather than robust evidence [7]. Moreover, they need to develop the evidence base for non-specialist models of palliative care due to the aforementioned (and probably ongoing) resource constraints. 

## 4. Models of Specialist Palliative Care

**Figure 1:**
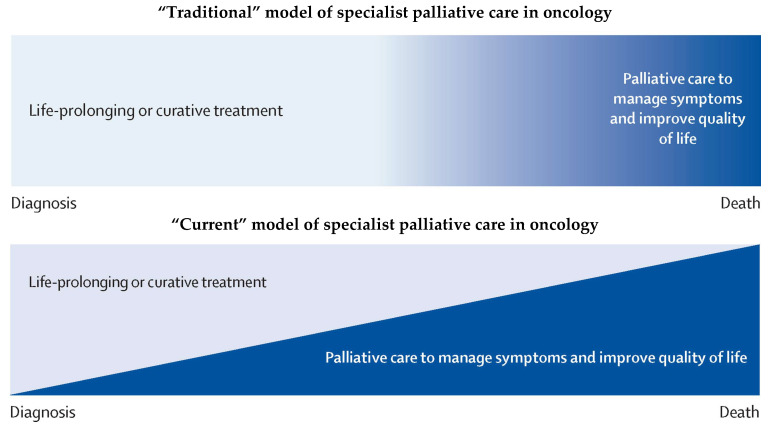
Traditional and current models of specialist palliative care in oncology (adapted from [8]).

As discussed, the “traditional” model of specialist palliative care focussed on patients with advanced cancer and involved both symptom control and end-of-life care (Figure 1) [8]. The “current” model of specialist palliative care reiterates the relevance of end-of-life care, but also highlights the importance of symptom controlin other groups of patients with cancer from the point of diagnosis. The model suggests the steadily increasing involvement of specialist palliative care, whereas, in reality, such involvement is much more variable. For example, many patients with early disease require “one-off” or short-lived input for symptom control and then no further involvement from the specialist palliative care team (unless they go on to develop advanced disease). 

Specialist palliative care services for patients with cancer are extremely heterogenous in terms of the services provided and their accessibility or availability to different groups of patients with cancer. Hospital-based services include outpatient clinics, inpatient reviews (i.e., the specialist palliative care team supporting the primary clinical team), and inpatient management (i.e., the specialist palliative care team becoming the primary clinical team). Similarly, community-based services include outpatient clinics, home reviews, and inpatient management. It should be noted that community-based services are generally restricted to patients with more advanced disease, especially patients no longer receiving curative anticancer treatment.

Almost invariably, specialist palliative care is provided by multidisciplinary teams, although the composition of the multidisciplinary team is again extremely heterogeneous. The “core” (essential) members include physicians and nurses, whilst some guidelines also suggest the addition of social workers and pastoral care personnel [9]. The extended body of members includes (but is not limited to) psychologists, social workers, pastoral care personnel, pain specialists, physiotherapists, occupational therapists, dieticians, and pharmacists [9].

Limited resources (human, financial, etc.) constitute a major factor in the suboptimal provision of specialist palliative care services worldwide. In response, the ASCO has produced resource-stratified guidelines on the provision of palliative care (ranging from “basic”, through “limited” and “enhanced”, to “maximal”), which address issues such as models of care, clinical staffing (and roles), access to social work/counselling, access to spiritual care, and the availability of opioid analgesics [10,11].

## 5. Evidence of Specialist Palliative Care in Oncology

Unsurprisingly, most of the evidence for specialist palliative care in oncology relates to patients with advanced cancer. In 2017, an ASCO Expert Panel published an updated Clinical Practice Guideline on the integration of palliative care into oncology, with a specific focus on patients with advanced cancer (i.e., “those with distant metastases, late-stage disease, cancer that is life limiting, and/or with prognosis of 6 to 24 months”) [1]. The expert panel reviewed the literature and provided a series of recommendations based on a series of clinical questions (Box 2). The key recommendation was that “patients with advanced cancer, whether inpatient or outpatient, should receive dedicated palliative care services, early in the disease course, concurrent with active treatment” (i.e., so-called “early palliative care”). Importantly, the expert panel noted that there was no evidence of unfavourable clinical outcomes with early palliative care and that there was some evidence of health-related economic benefits (and no evidence of health-related economic detriments).

Box 2 **ASCO expert panel’s recommendations for the integration of palliative care into oncological services.**

***Clinical question: What is the most effective way to care for patients with advanced-cancer symptoms (palliative care services in addition to usual care compared to usual care alone)?***

Recommendation: Patients with advanced cancer should be referred to interdisciplinary palliative care teams (consultation) that provide inpatient and outpatient care early in the course of disease, alongside active treatment of their cancer **(type: evidence-based and benefits outweigh harms; evidence quality—intermediate; strength of recommendation—strong)**.

 

***Clinical question: What are the most practical models of palliative care? Who should deliver palliative care (external consultation, internal consultations with palliative care practitioners in the oncology practice, or by an oncologist him- or herself)?***

Recommendation: Palliative care for patients with advanced cancer should be delivered through interdisciplinary palliative care teams, with consultation available in both outpatient and inpatient settings **(type: evidence-based and benefits outweigh harms; evidence quality—intermediate; strength of recommendation—moderate)**.

 

***Clinical question: How is palliative care in oncology defined or conceptualized?***

Patients with advanced cancer should receive palliative care services, which may include a referral to a palliative care provider. The essential components of palliative care include rapport and relationship building with patient and family caregivers; symptom, distress, and functional status management (e.g., with respect to pain, dyspnoea, fatigue, sleep disturbance, mood, nausea, or constipation); exploration of understanding and education about illness and prognosis; clarification of treatment goals; assessment and support of coping needs (e.g., provision of dignity therapy); assistance with medical decision making; coordination with other care providers; and the provision of referrals to other care providers, as indicated. For newly diagnosed patients with advanced cancer, the expert panel suggests early palliative care involvement, starting early in the diagnosis process and ideally within 8 weeks of diagnosis (**type: informal consensus; evidence quality—intermediate; strength of recommendation—moderate)**.

 

***Clinical question: How can palliative care services relate in practice to other existing or emerging supportive care services (including nurse navigation, lay navigation, community and home health care, geriatric oncology, psycho-oncology, and pain services)?***

Among patients with cancer with a high symptom burden and/or unmet physical or psychosocial needs, outpatient programs of cancer care should provide and use dedicated resources (palliative care clinicians) to deliver palliative care services to complement existing program tools **(type: informal consensus and benefits outweigh harms; evidence quality—intermediate; strength of recommendation—moderate)**.

 

***Clinical question: Which interventions are helpful for family caregivers?***

For patients with early or advanced cancer for whom family caregivers will provide care in outpatient, home, or community settings, nurses, social workers, or other providers may initiate caregiver-tailored palliative care support, which could include telephone coaching, education, referrals, and face-to-face meetings. For family caregivers who may live in rural areas and/or be unable to travel to a clinic and/or longer distances, telephone support may be offered **(type: evidence-based; evidence quality—low; strength of recommendation—weak)**.

## 6. Triggers for Referral to Specialist Palliative Care Services

In cancer services, the usual criteria for referral to specialist palliative care services are the presence of advanced cancer and/or the presence of uncontrolled cancer-related symptoms (or other relevant unmet needs). Importantly, the aforementioned ASCO expert panel identified a number of other primary (“global”) and secondary (“more specific”) triggers for a specialist palliative care assessment (Box 3), which may or may not result in further specialist palliative care intervention [1].

Box 3 **Criteria for specialist palliative care assessment (adapted from [1]).**

Primary triggers (global indicators):

▘ “Surprise question”: you would not be surprised if the patient died within 12 months;
▘ Admission prompted by difficult-to-control physical or psychological symptoms;
▘ Decline in function;
▘ Feeding problems or unintended weight loss;
▘ Frequent admissions;
▘ Complex care requirements.

Secondary triggers (specific indicators):

▘ Locally advanced or metastatic cancer;
▘ Elderly patient;
▘ Cognitively impaired;
▘ Admission from long-term care facility;
▘ Limited social support.

Several tools may prompt referral to specialist palliative care services. For example, generic and disease-specific indicators within the Supportive and Palliative Care Indicators Tool (SPICT) may aid the identification of adult patients at risk of deterioration and dying and those with unmet needs, who may benefit from the holistic approach provided by specialist palliative care [12,13]. For patients with cancer, this includes progressive cancer resulting in functional decline, anticancer treatment for symptom control, or frailty precluding anticancer therapies [12].

## 7. Specialist Palliative Care versus Supportive Care

The term “supportive care” has been used to describe various interventions or strategies for patients with cancer [14]. However, the Multinational Association of Supportive Care in Cancer (MASCC) defines supportive care as “the prevention and management of adverse effects of cancer and its treatment. This includes management of physical and psychological symptoms and side effects across the continuum of the cancer journey from diagnosis through treatment to post-treatment care” [15].

**Figure 2:**
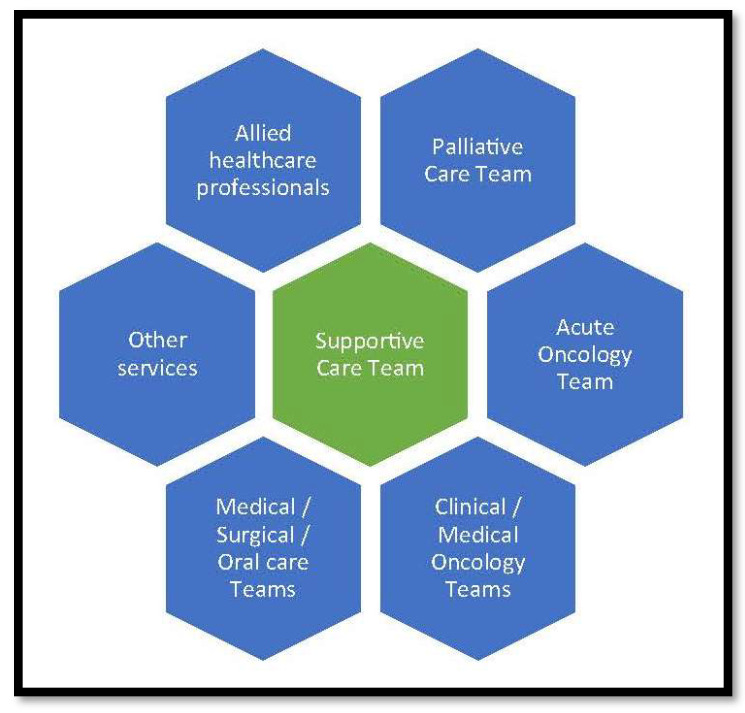
The “extended” supportive care team [16].

Supportive care has been used as a synonym for palliative care (especially early palliative care). However, although palliative care is an integral component of supportive care, supportive care is much more than palliative care and should involve input from a range of specialist teams and services (Figure 2) [16]. Furthermore, specialist palliative care services are unlikely to be able to meet all supportive care needs, especially since many have limited knowledge and experience in managing the adverse effects of anticancer treatment, particularly those of newer immunotherapies or targeted therapies [17].

Nevertheless, several specialist palliative care services have re-branded themselves as supportive care services (or supportive and palliative care services). This has resulted from the negative professional and public perceptions regarding the affiliation of palliative care with end-of-life care and the concern about causing a loss of hope. Research suggests that such a name change can lead to an increase in referrals from oncology healthcare professionals, and specifically referrals of patients at an earlier stage than beforehand [18]. 

## 8. Specialist Palliative Care and Cancer Survivorship

The National Cancer Institute (USA) defines a cancer survivor as follows: “a person is considered to be a survivor from the time of diagnosis until the end of life” [19]. This definition is wide ranging and includes cohorts of patients that are usually referred to specialist palliative care services, i.e., patients with advanced cancer and patients with acute symptoms relating to their cancer/anticancer treatment. However, the definition also includes cohorts of patients that are generally not referred to specialist palliative care services, i.e., patients with stable cancer on “maintenance” anticancer treatment, patients without active cancer, and patients with chronic symptoms resulting from previous anticancer treatment.

Specialist palliative care teams are experienced in managing the acute problems associated with cancer (and to a lesser extent cancer treatment, as previously explored), but are less experienced in managing the chronic problems observed among “long-term” cancer survivors. Importantly, interventions that are effective in treating acute problems may be less effective in managing chronic problems and may be less appropriate or even inappropriate due to the required duration of treatment (i.e., long-term or indefinite) [17]. Moreover, certain chronic problems necessitate highly specialised investigation and management [20,21].

Chronic pain is common among long-term cancer survivors and is often related to a preceding anticancer treatment. Specialist palliative care services are adept at managing acute cancer pain, and the cornerstone of treatment is a variety of pharmacological interventions (e.g., non-opioids, opioids, adjuvant analgesics, and combination therapies). Opioid analgesics are very effective in managing acute pain, but they are generally less effective in managing chronic pain [22]. Moreover, there are concerns about the long-term effects of opioids on the endocrine system (hypogonadism), the immune system, the respiratory system, and the central nervous system, as well as the perennial risk of drug misuse and abuse [23]. It should be noted that chronic pain is generally managed by pain teams and that the focus of care is on non-pharmacological interventions (e.g., therapeutic exercise, cognitive behavioural therapy, etc.) rather than pharmacological interventions.

## 9. Conclusions

All patients with cancer, at whatever stage and with whatever prognosis, should be treated using a palliative care approach to ensure optimal quality of life (a palliative care approach and general palliative care). However, patients with advanced cancer, and patients with difficult-to-manage cancer-related symptoms, should be referred in a timely fashion to specialist palliative care services since the benefits of referral are now well established (and recommended in national and international oncology guidelines).
